# Supplementary material for: TNF/IFN-γ Co-Signaling Induces Differential Cellular Activation in COVID-19 Patients: Implications for Patient Outcomes
Source: Int J Mol Sci. 2025 Jan 28;26(3):1139. doi: 10.3390/ijms26031139 (PMC11817726; doi:10.3390/ijms26031139)
Supplement: Supplementary file 1 [file ijms-26-01139-s001.zip › ijms-3410474-supplementary.pdf]

International Journal of *Molecular Sciences*

MDPI

*Article*

# The TNF/IFN-γ co-signaling induces a differential cellular activation in COVID-19 patients: Implications for patient outcomes.

**Lucero A. Ramón-Luing[1], Laura E. Martínez-Gómez[2], Carlos Martinez-Armenta[2], Gabriela A. Martínez-Nava[2], Karen Medina-Quero[3], Gloria Pérez-Rubio[1], Ramcés Falfán-Valencia[1], Ivette Buendia-Roldan[1], Julio Flores-Gonzalez[1], Ranferi Ocaña-Guzmán[1], Moisés Selman[1], Alberto López-Reyes[2], Leslie Chavez-Galan[1]***

[1] Instituto Nacional de Enfermedades Respiratorias Ismael Cosío Villegas, Mexico City, 14080, Mexico. ramonluing@yahoo.com.mx (LARL), glofos@yahoo.com.mx (GPR), dcb_rfalfanv@hotmail.com (RFV), ivettebu@yahoo.com.mx (IBR), juliofglez@gmail.com (JFL), arocana@iner.gob.mx (ROG), mselmanl@yahoo.com.mx (MS).

[2] Laboratorio de Gerociencias, Instituto Nacional de Rehabilitación Luis Guillermo Ibarra Ibarra, Mexico City, 14080, Mexico. laurae.mtzg@gmail.com (LEMG), c.armenta1208@gmail.com (CMA), ameria.justice@gmail.com (GAMN), allorey@yahoo.com (ALR).

[3] Immunology laboratory, Escuela Militar de Graduados de Sanidad, Mexico City, 11200, Mexico. kmq.kmq5@gmail.com (KMQ).

* Correspondence: Leslie Chávez-Galán, PhD
Laboratorio de Inmunología Integrativa, Instituto Nacional de Enfermedades Respiratorias Ismael Cosio Villegas. Email: lchavezgalan@gmail.com; lchavez_galan@iner.gob.mx

**Citation:** To be added by editorial staff during production.

Academic Editor: Firstname Lastname

Received: date
Revised: date
Accepted: date
Published: date

**Copyright:** © 2024 by the authors. Submitted for possible open access publication under the terms and conditions of the Creative Commons Attribution (CC BY) license (https://creativecommons.org/licenses/by/4.0/).

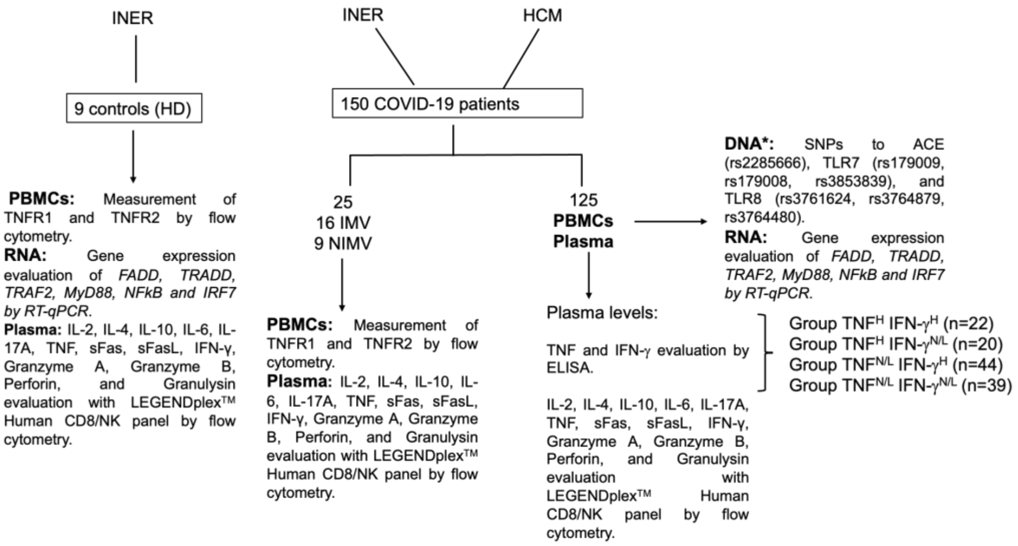


**Supplementary Figure S1**. Workflow of enrolled patients. One hundred fifty-nine subjects were recruited from the National Institute of Respiratory Diseases (INER) and Military Central Hospital (HCM). Nine subjects clinically evaluated were considered healthy donors (HD, recruited from INER), and 150 with COVID-19 diagnosis (INER/HCM). From them, 25 were classified based on the use of invasive mechanical ventilation (IMV) or not (NIMV), and together with the HD, these groups were analyzed to cell phenotype by flow cytometry and plasmatic levels of soluble molecules IL-2, IL-4, IL-10, IL-6, IL-17A, TNF, sFas, sFasL, IFN-γ, Granzyme A, Granzyme B, Perforin, and Granulysin using LEGENDplex™ Human CD8/NK panel by flow cytometry. From another 125 COVID-19 patients, the evaluation of single nucleotide polymorphisms (SNPs) and the transcriptional level of molecules related to TLR signaling, TNF, IFN-γ, and soluble molecules were evaluated. Plasma TNF and IFN-γ levels were used to classify COVID-19 patients as patients with a profile TNF$^{H}$ and IFN-$\gamma^{H}$, TNF$^{H}$ and IFN-$\gamma^{N\text{-}L}$, TNF$^{N\text{-}L}$IFN-$\gamma^{H}$, and TNF$^{N\text{-}L}$IFN-$\gamma^{N\text{-}L}$. High (H) or normal-low (N-L) levels were determined considering the mean value reported to HD (cutoff values of 10.2 pg/mL for TNF and 12.6 pg/mL for IFN-γ). * Three of the 125 subjects assigned to genotyping analysis were excluded because of the poor DNA concentration.

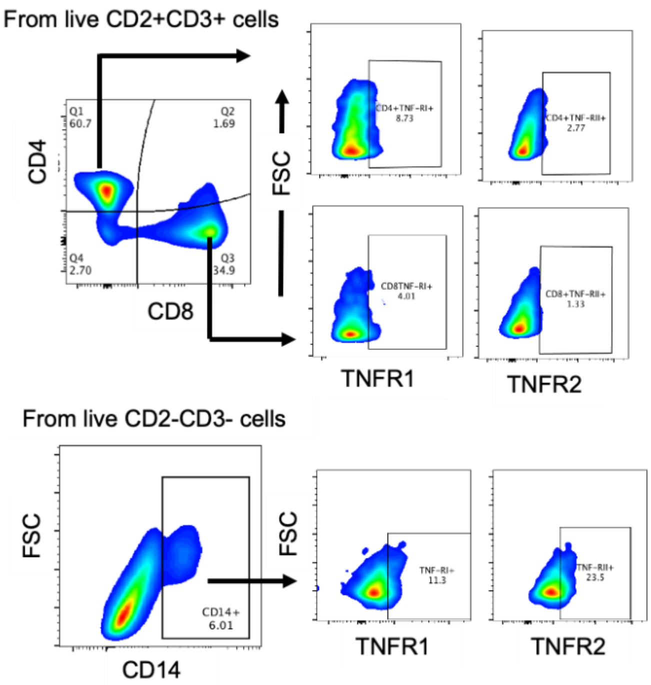


**Supplementary Figure S2.** Gating strategy to identify T cells and CD14+ monocytes. Representative pseudo-color plots illustrate the gating obtained by flow cytometry. Single viable cells were selected from peripheral blood mononuclear cells. For T cells, the CD2+CD3 gate was set, and the coexpression of CD4 and CD8 was identified (up). For monocytes, CD2+ and CD3+ cells were excluded, and then the CD14 expression was identified (down). Within the gates of CD4+, CD8+ T cells, and monocytes, the coexpression of TNFR1 and TNFR2 was identified.

**Table S1.** Baseline characteristics of COVID-19 patients who received invasive mechanical ventilation or not.

| | **IMV (n=16)** | **NIMV (n=9)** | ***P**** |
|---|---|---|---|
| Age (years) | 53 | 48 | 0.7918 |
| | (44-59) | (36-75) | |
| **Sex** | | | |
| Male, n (%) | 8 (50) | 8 (89) | 0.0518 |
| Female (%) | 8 (50) | 1 (11) | |
| Leukocyte, x$10^9$/L | 11.9 | 9.8 | 0.3497 |
| | (8.82-14.33) | (7.9-13.4) | |
| D-dimer (ng/mL) | 2.065 | 0.48 | 0.0133 |
| | (0.99-4.65) | (0.30-1.45) | |
| LDH (U/L) | 518.0 | 331.0 | 0.0028 |
| | (384.8- 609.8) | (249.5- 346) | |

Data are presented as number (percentage) or median (interquartile range). LDH, lactate dehydrogenase. *$p$ values were calculated using the chi-squared and the U de Mann Whitney Test for comparisons between the two groups of COVID-19 patients who received invasive mechanical ventilation (IMV) or not (NIMV) and tested for TNFR1 and TNFR2 expression.

**Table S2.** Allelic frequencies among the groups of study.

| **SNP** | **Total** | **Group 1** | **Group 2** | **Group 3** | **Group 4** | ***P* value*** |
|---|---|---|---|---|---|---|
| **rs2285666** | | | | | | |
| C | 147 (60%) | 19 (45%) | 22 (55%) | 55 (64%) | 51 (67%) | 0.09 |
| T | 97 (40%) | 23 (55%) | 18 (45%) | 31 (36%) | 25 (32%) | |
| **rs179009** | | | | | | |
| A | 165 (67%) | 28 (67%) | 26 (65%) | 58 (67%) | 53 (70%) | 0.96 |
| G | 79 (32%) | 14 (33%) | 14 (35%) | 28 (33%) | 23 (30%) | |
| **rs179008** | | | | | | |
| A | 213 (87%) | 36 (86%) | 38 (95%) | 71 (83%) | 68 (89%) | 0.23 |
| T | 31 (13%) | 6 (14%) | 2 (5%) | 15 (17%) | 8 (11%) | |
| **rs2302267** | | | | | | |
| G | 222 (91%) | 39 (93%) | 36 (90%) | 80 (93%) | 67 (88%) | 0.71 |
| T | 22 (9%) | 3 (7%) | 4 (10%) | 6 (7%) | 9 (12%) | |
| **rs3853839** | | | | | | |
| G | 151 (62%) | 12 (29%) | 31 (77%) | 56 (65%) | 52 (68%) | **<0.001** |
| C | 93 (38%) | 30 (71%) | 9 (22%) | 30 (35%) | 24 (32%) | |
| **Haplotype** | | | | | | |
| ACA | 74(37%) | 16(28%) | 26(65%) | 8(22%) | 24(36%) | **<0.001** |
| GGG | 124(63%) | 40(71%) | 14(35%) | 28(78%) | 42(64%) | |

*Chi-squared test.

Three of the 125 subjects assigned to genotyping analysis were excluded because of the poor DNA concentration; thus, 122 were genotyped, 84 men and 38 women.

Values in bold denote statistically significant.

**Table S3.** TNF/IFN-γ levels and TLR7/TLR8 polymorphism stratified by sex.

| | | | | | | |
|---|---|---|---|---|---|---|
| | | | **Men** | | | |
| | **TNF levels** | | | **IFN-γ levels** | | |
| **Polymorphism** | **Ancestral** | **Variant** | **P value*** | **Ancestral** | **Variant** | **P value*** |
| **rs2285666** | 6<br>(0.55-12.3) | 10.745<br>(7.06-16.8) | **0.006**** | 10.3<br>(0.25-37) | 15<br>(5.7-32.35) | 0.098** |
| **rs179009** | 7.475<br>(0.55-14.13) | 8.675<br>(1.38-15.7) | 0.390** | 15.915<br>(2.6-35.205) | 10.215<br>(0.25-30.98) | 0.287** |
| **rs179008** | 7.9<br>(0.55-14.13) | 6.58<br>(2.52-27.45) | 0.770** | 12.2<br>(1.55-32.88) | 27.385<br>(5.99-76.25) | 0.181** |
| **rs2302267** | 7.85<br>(0.55-14.13) | 7.625<br>(0.82-17.35) | 0.897** | 15.71<br>(2.05-35.58) | 6.75<br>(0.37-12.66) | 0.069** |
| **rs3853839** | 6.93<br>(0.55-12.3) | 9.865<br>(3.85-15.7) | 0.107** | 9.35<br>(0.25-56.55) | 23.65<br>(3.5-42.66) | **0.006**** |
| **Haplotypes TLR8** | 9.9<br>(4.9-15.7) | 23.7<br>(3.15-41.77) | 0.06** | 23.7<br>(3.15-41.77) | 11.2<br>(0.6-26.97) | 0.04 |
| | | | **Woman** | | | |
| | **TNF levels** | | | **IFN-γ levels** | | |
| **Polymorphism** | **Ancestral** | **Variant** | **P value*** | **Ancestral** | **Variant** | **P value*** |
| **rs2285666** | 7.8<br>(4.5-10.2) | 6.58<br>(0.55-10.1) | 0.516** | 21.08<br>(5-58) | 21.5<br>(1.1-63.65) | 0.995** |
| **rs179009** | 7.8<br>(5.6-11.2) | 3.85<br>(0.55-8.51) | 0.010** | 11.2<br>(0.25-60.61) | 35.15<br>(21.08-60.61) | 0.083** |
| **rs179008** | 6.8<br>(4.5-11.2) | 3.85<br>(0.55-8.51) | 0.086** | 15.81<br>(1.1-63.65) | 33.84<br>(11.4-57.9) | 0.519** |
| **rs2302267** | 6.7<br>(3.85-10.2) | 7.255<br>(4.35-9) | 0.915** | 21.08<br>(3.6-60.61) | 39.145<br>(13.8-60.61) | 0.564** |
| **rs3853839** | 6.8<br>(3.4-10.1) | 6.58<br>(4.35-15.43) | 0.663** | 15.81<br>(3.6-39.99) | 38.3<br>(3.6-77.14) | 0.282** |
| **Haplotypes TLR8** | 5.6<br>(0.55-15.43) | 8.51<br>(0.55-11.2) | 0.49** | 57.9<br>(33.84-60.61) | 11.4<br>(33.84-60.61) | 0.02** |

Due to the localization of the genes in X chromosomes, a sex-stratified analysis of soluble levels of IFN-γ and TNF was performed.

Cytokine levels (pg/mL) are shown as median with interquartile range (IQR).

*U de Mann Whitney Test. Values in bold denote statistically significant. **P adjusted by Bonferroni correction.

**Table S4.** Monoclonal antibodies used for flow cytometry.

| Antibody | Fluorochrome | Brand | Clone | Isotype | Catalog |
| --- | --- | --- | --- | --- | --- |
| anti-CD14 | Brilliant Violet 510 | BioLegend | M5E2 | Mouse IgG2a, κ | 301842 |
| anti-CD2 | APCH7/APCCY7 | BioLegend | TS1/8 | Mouse IgG1, κ | 309238 |
| anti-CD3 | Brilliant Blue 700 | BD Bioscience | SK7 | Mouse IgG1, κ | 566575 |
| anti-CD4 | Brilliant Violet 510 | BioLegend | T4/Leu-3 | Rat IgG2b, κ | 357420 |
| anti-CD8 | Brilliant Ultraviolet 563 | BD Bioscience | RPA-T8 | Mouse IgG1, κ | 612914 |
| anti-TNF-RI | APC | BioLegend | W15099A | Mouse IgG2a, κ | 369906 |
| anti-TNF-RII | PE/Dazzle 594 | BioLegend | 3G7A02 | Rat IgG2a, κ | 358414 |

**Disclaimer/Publisher's Note:** The statements, opinions and data contained in all publications are solely those of the individual author(s) and contributor(s) and not of MDPI and/or the editor(s). MDPI and/or the editor(s) disclaim responsibility for any injury to people or property resulting from any ideas, methods, instructions or products referred to in the content.
